# Meta-analysis of the effects of overexpression of WRKY transcription factors on plant responses to drought stress

**DOI:** 10.1186/s12863-019-0766-4

**Published:** 2019-07-26

**Authors:** Yuan Guo, Wenjing Ping, Jingtang Chen, Liying Zhu, Yongfeng Zhao, Jinjie Guo, Yaqun Huang

**Affiliations:** Hebei Branch of Chinese National Maize Improvement Center, Hebei Agricultural University, Baoding, People’s Republic of China

**Keywords:** Drought, WRKY transcription factors, Over-express, Meta-analysis

## Abstract

**Background:**

The tryptophan-arginine-lysine-tyrosine (WRKY) transcription factors play important roles in plants, allowing them to adapt to environmental conditions that are not normally conducive to plant growth; in particular, drought. There has been extensive research on WRKY transcription factors and the effects of their overexpression in plants on resistance to drought stress. However, due to the materials (the type and species of donor and receptor, promoters) and treatments (the type and time of stress) used, different and often confounding results have been obtained between studies. Meta-analysis is a powerful statistical tool that can be used to summarize results from numerous independent experiments on the same research topic while accounting for variability across experiments.

**Results:**

We carried out a meta-analysis of 16 measured parameters that affect drought resistance in plants overexpressing WRKY transcription factors and wild-type plants. We found that only one of these parameters was significantly different between transgenic and wild-type plants under drought and control conditions at a 95% confidence interval (*p* = 0.000, *p* = 0.009, respectively). Eleven of the sixteen parameters were obviously different in WRKY transgenic plants under drought and control conditions (SV, *p* = 0.023, SSC, *p* = 0.000, SOD, *p* = 0.012, SFW, *p* = 0.000, RL, *p* = 0.016, Pro, *p* = 0.000, POD, *p* = 0.027, MDA, *p* = 0.000, H_2_O_2_, *p* = 0.003, EL, *p* = 0.000, CHC, *p* = 0.000, respectively), seven of the eleven obviously different parameters showed positive effect (SSC, SOD, Pro, POD, MDA, H_2_O_2_, EL), four of them revealed negative effect (SV, SFW, RL, CHC).

**Conclusion:**

We have found that only one of these parameters was significantly different between transgenic and wild-type plants under drought and control conditions respectively, at a 95% confidence interval. And eleven of sixteen parameters showed obviously different of WRKY-overexpressed plants under different conditions (water-stressed and normal), suggesting that WRKY transcription factors play an important role in plant responses to drought stress. These findings also provide a theoretical basis for further study of the role of WRKY transcription factors in the regulation of plant responses to environmental stress.

**Electronic supplementary material:**

The online version of this article (10.1186/s12863-019-0766-4) contains supplementary material, which is available to authorized users.

## Background

Drought is a type of dehydration stress and largely restricts crop productivity and quality. Improving crop tolerance to drought stress is an effective strategy for the promotion of sustainable agriculture in most crop production regions worldwide [1]. There has been extensive research on plant responses to drought stress at the morphological [2–6], physiological [2, 7–15], and biochemical [16–21] levels. Almost 2000 drought-responsive genes have been identified in Arabidopsis under progressive soil drought stress [22]. Stress gene responses occur primarily at the transcription level; however, the temporal and spatial expression patterns of specific stress genes is an important aspect of the plant stress response [23].

A large number of transcription factors that are either up-or down-regulated in response to environmental perturbations have been identified using genome-wide transcriptome analyses [24, 25]. The tryptophan-arginine-lysine-tyrosine (WRKY) transcription factor family is one of the largest families of transcriptional regulators in plants and plays important roles in plant development and various stress responses [26]. Several studies have showed that WRKY transcription factors are involved in plant drought stress responses. For example, when Muhammad et al. overexpressed *CmWRKY10* from Chrysanthemum, the gene was highly up-regulated in transgenic Chrysanthemum under drought stress through the abscisic acid (ABA) signaling pathway [27]. Overexpression of the grape gene *VvWRKY11* in Arabidopsis seedlings increased tolerance to mannitol-induced water stress [28]. Wu et al. overexpressed *OsWRKY11* under the control of the HSP101 promoter in transgenic rice and found that it enhanced drought tolerance [29], with reduced leaf wilting and increased survival of green tissues. Overexpression of maize *ZmWRKY58* in rice increased the plant survival rate and relative water content (RWC), suggesting that *ZmWRKY58* overexpression enhances tolerance to drought and salt stresses in transgenic rice [30]. Similarly, Ding et al. [31] and Xu et al. [32] both overexpressed WRKY genes in wheat and found that the transgenic plants were more tolerant to water stress than wild-type (WT) plants. The important role played by WRKY transcription factors in plant resistance to drought stress has been increasingly verified across plant species. However, many of the results of these studies are confounded by differences in the species and treatments used. Therefore, there is a need to integrate a large number of studies to allow better interpretation of their results. In this study, we conducted a meta-analysis of the effects on WRKY transcription factors in plant responses to drought stress.

Meta-analysis is a powerful statistical tool that can be used to summarize results from multiple independent experiments while accounting for variability across experiments [33]. The applications of meta-analytical approaches are extensive. Meta-analysis has been used to examine the responses of different crop species to biotic and/or abiotic stresses; for example, heat stresses in nitrogen pools. It has also been widely used to examine plant adaptions to abiotic stress. Wang et al. used a meta-analysis of published data to quantitatively evaluate the effects of drought stress on the morphophysiological and biochemical characteristics, growth and biomass partitioning, and yield formation of different wheat ploidies [34]. They found that domestication and selection of higher ploidy wheat has reduced the adverse effects of drought stress on yield and yield components, optimized biomass allocation toward higher seed yields, and reduced stress-related physiological and biochemical responses. Ma et al. [35] and Augé et al. [36] used meta-analyses to explore responses to NaCl stress; namely, the regulation of the main cation/proton antiporter gene and arbuscular mycorrhizal symbiosis, respectively. These studies provided a reference for further investigations of plant responses to salt stress. Dong et al. conducted a meta-analysis of plants overexpressing DREB transcription factors and found that of 13 measured parameters, 8 and 2 exhibited significant transcriptional responses in drought-stressed and control plants, respectively.

When plants are subjected to drought stress, they respond by adapting to the environmental conditions. The comprehensive response to drought that occurs in plants encompasses changes to their morphology and anatomy, and physiological and biochemical modifications that occur at many different levels; ranging from individual cells, to photosynthetic organs, to the entire structure of the plant [37]. An overall assessment of drought tolerance can be determined by measuring plant growth and yield; however, specific morphological, anatomical, physiological, and biochemical parameters can also serve as indicators of plant responses to stress [38]. In this study, we used an integrated analysis of 16 physiological and morphological plant parameters that were measured across a relatively large number of studies. These included the plant survival rate, soluble sugar content, superoxide dismutase (SOD) activity, peroxidase (POD) activity, catalase (CAT) activity, H_2_O_2_ content, shoot fresh weight, stomatal aperture, RWC, root length, proline content, plant height, germination rate, electrolyte leakage, chlorophyll content and malondialdehyde content.

## Results

### Summary of overall effects

**Fig. 1:**
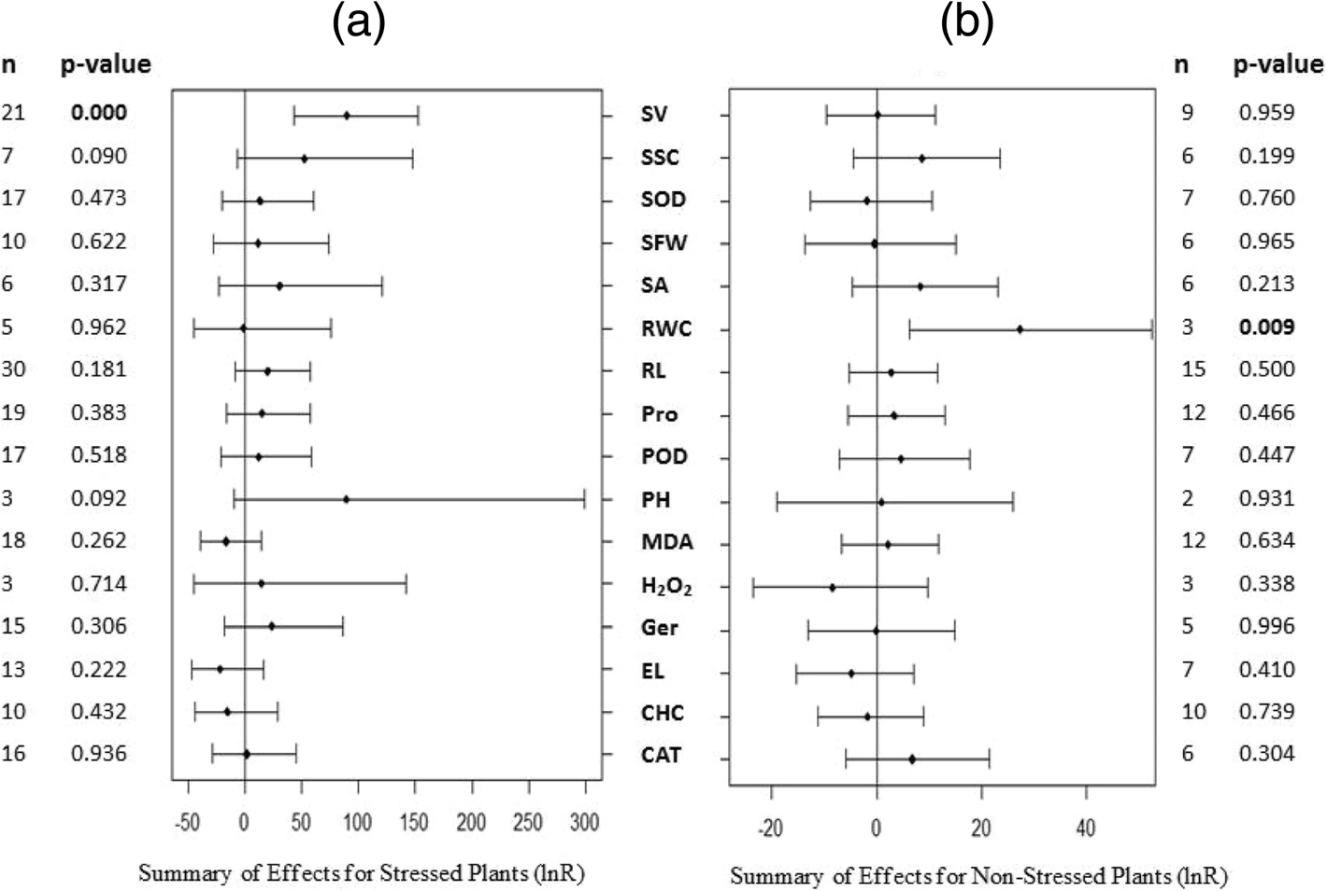
Summary of effects of drought resistance parameters for WRKY-overexpressed and WT plants subject to drought stress (**a**) and non-stressed conditions (**b**) Summary of weighted effects (ln R, natural logarithm of the WRKY-overexpressed/WT plants response ratio). Summary of effects are shown in filled dots and horizontal bars show the 95% confidence interval. n, the number of studies for each summary effect numbers in boldindicate significant difference (*P* ≤ 0.05) (same for Figs. 2, 3, 4, 5, 6, 7, 8)

Our meta-analysis included 158 studies published in 47 journal articles. These contained 19 species, including 6 monocots and 13 dicots. 5 gramineous plants, 3 legume plants, 2 solanaceae plants, 2 compositae plants, and 9 other types of plants. A number of the response variables commonly measured in plants as adaptations to drought stress were listed in Fig. 1. The cauliflower mosaic virus CaMV35S promoter was most widely used (109 studies). The two most commonly appeared recipient species were Arabidopsis and tobacco (69 and 58 studies, respectively), and almost all were dicots (153 studies). Most of the studies introduced a single foreign WRKY gene (140 studies), although some introduced two genes (18 studies). Two-thirds of the gene donor species were dicots (96 studies).

One of the 16 measured plant parameters was obviously different between transgenic and WT plants subjected to drought stress conditions at a 95% confidence interval (survival rate; Fig. 1a). Three parameters were significantly or close to significantly different at a 90% confidence interval (root length, plant height and soluble sugar content; Fig. 1a). Under non-stressed conditions, 1 of the 16measured plant parameters was significant at a 95% confidence interval (RWC), and 1 parameter was obviously different at a close-to-90% confidence interval (soluble sugar content; Fig. 1b).

**Fig. 2:**
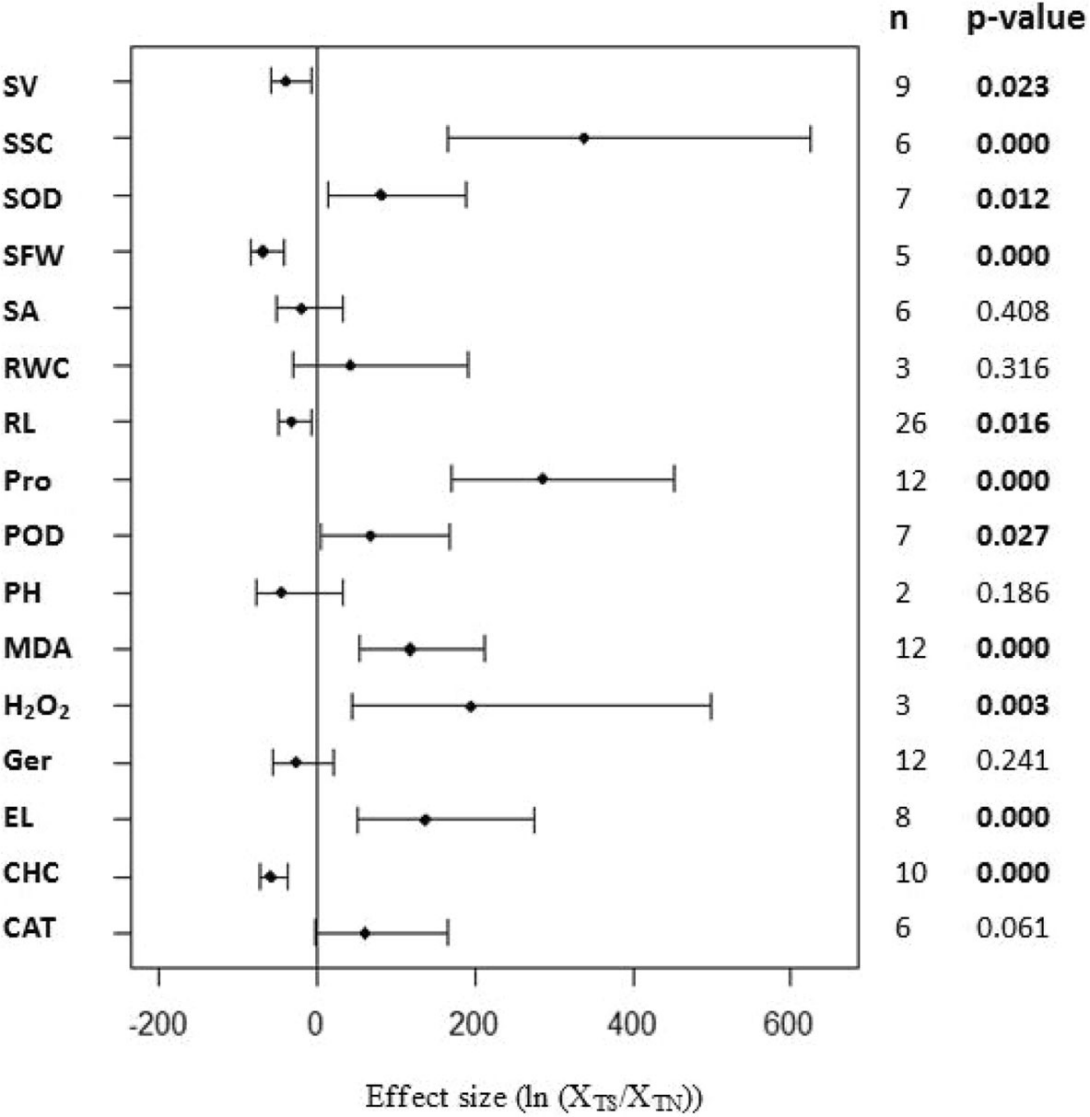
Summary of effects of drought resistance parameters for WRKY-overexpressed plants under drought-stressed/non-stressed conditions. Weighted summary effect sizes (ln (X_TS_/X_TN_), natural logarithm of the WRKY-overexpressed plants under stressed /non-stressed conditions response ratio; X_TS_, the means of WRKY-overexpressed plants under stressed conditions; X_TN_, the means of WRKY-overexpressed plants under non-stressed conditions)

To better evaluate the role of WRKY transcription factors in plant resistance to drought stress, we compared transgenic plants under drought and normal conditions (Fig. 2). Eleven of the 16 measured plant parameters had significant differences (*p* ≤ 0.05): 7 parameters were increased with drought treatment (soluble sugar content, proline content, malondialdehyde content, electrolyte leakage, H_2_O_2_content, SOD activity and POD activity) and 4 parameters were reduced (survival rate, shoot fresh weight, root length and chlorophyll content). Among of them, the soluble sugar content of transgenic plants changed most (339.1%) under drought stressed environment, compared with non-stressed condition.

### Subgroup analysis of the survival rates of plants under drought conditions

**Fig. 3:**
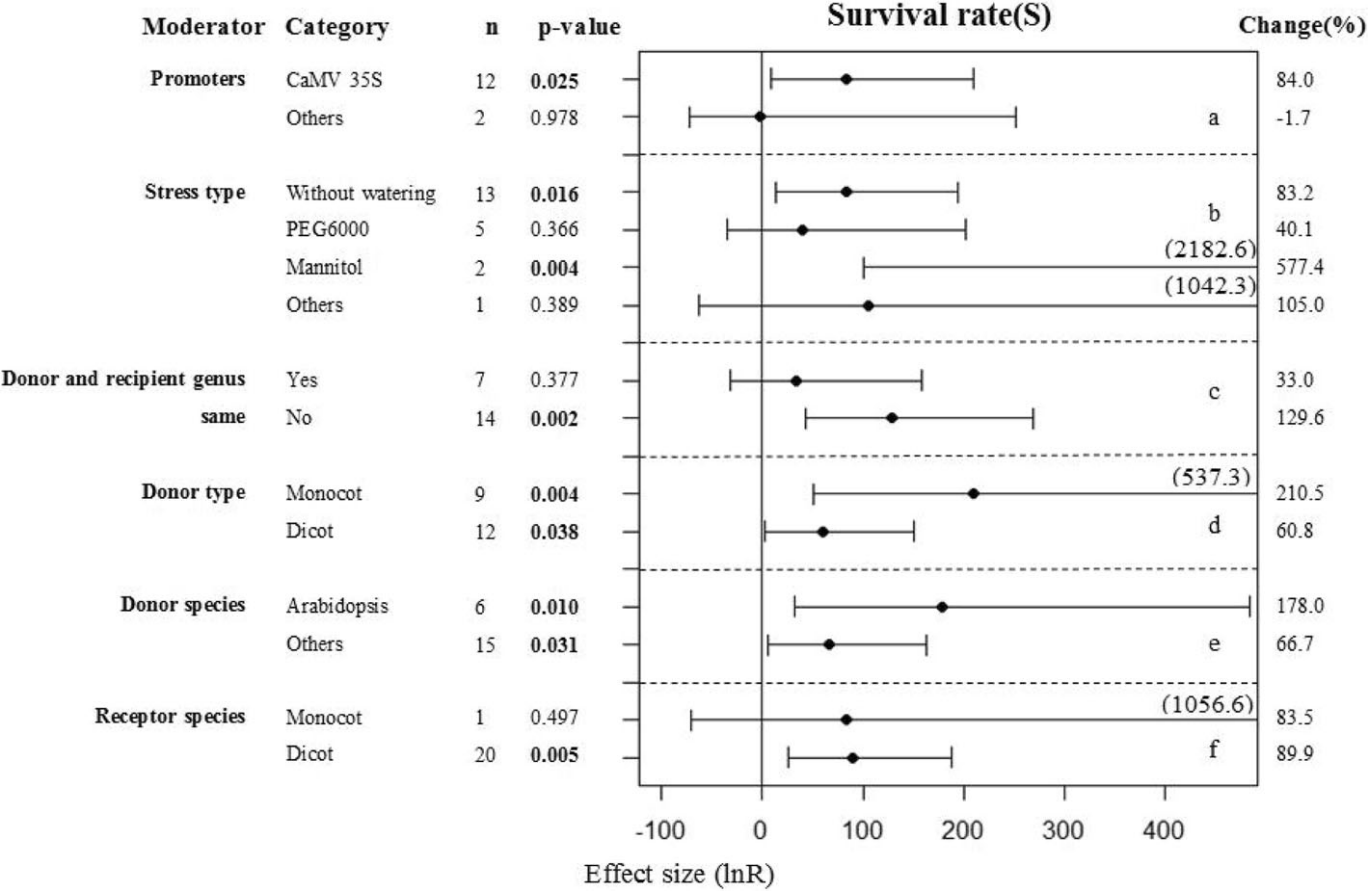
Subgroup analysis of the effects on survival rate of plants under drought-stressed condition. There are six moderators on survival rate in plants subject to drought (**a**-**f**) and category represents each moderator level

The specific effects of overexpressing WRKY genes on survival rates of plants subjected to drought conditions were significant when WRKY expression was driven by the CaMV35S promoter (Fig. 3). Different type of stress also contributed to the results, when transgenic plants treated with application of mannitol and without watering, it had significantly enhanced survival rates. If the gene donor and recipient species are from two different genera, it was hypothesized that overexpression of WRKY transcription factors might increase the survival rate. However, the species and type of gene donor had little effect. When the recipient species was a dicot, the transgenic plants showed improved survival. While due to the small number of studies included, there was only one study used monocot species as recipient and effect sizes resulted too large confidence intervals and a low level of precision (Fig.3f).

### Subgroup analysis of the root lengths of plants under drought conditions

**Fig. 4:**
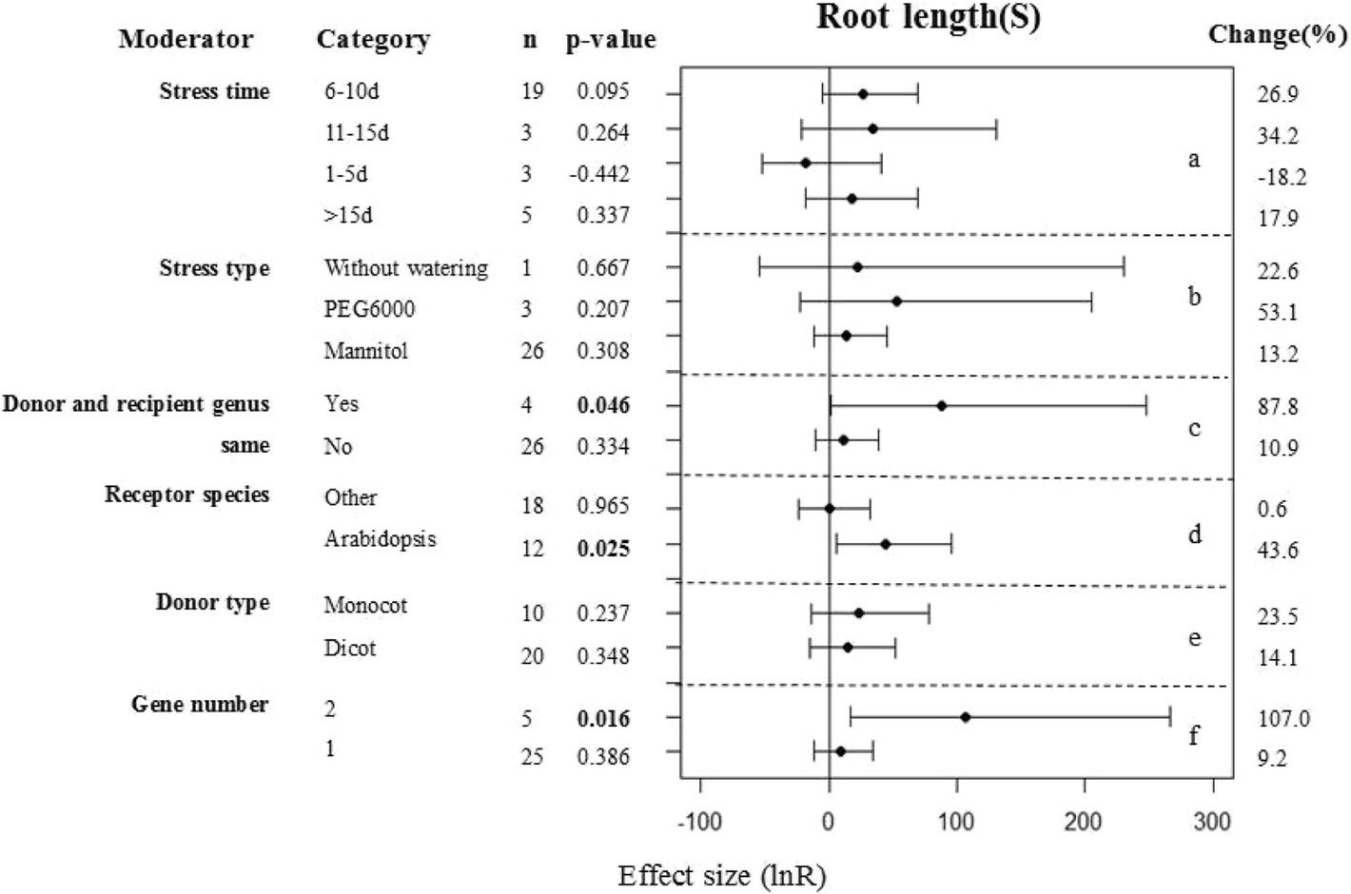
Subgroup analysis of the effects on root length of plants under drought-stressed condition. There are six moderators on root length in plants subject to drought (**a**-**f**) and category represents each moderator level

The transgenic plants did not show any significant differences in their root lengths under drought conditions at a 95% confidence interval (*p* = 0.181; Fig. 1). However, the confidence interval was close to 90%, indicating an almost 90% confidence that WRKY transcription factors enhanced the root lengths of transgenic plants when water was in limited supply. Because the root length sample size was relatively large, we were able to further explore the factors affecting WRKY transcription factor expression and the root lengths of these transgenic plants (Fig. 4). Different exposure times and types of stress did not affect the results (Fig. 4a, b). A significantly greater positive moderator effect was observed when the gene donor and recipient were from the same genus than when the donor and recipient were from the different genus.(Fig. 4c). When the recipient was from a species other than Arabidopsis, the transgenic plants had significantly increased root lengths (Fig. 4d). When the transgenic plants had two WRKY genes introduced, their beneficial effects on root lengths were more pronounced (Fig. 4f). However, the type of donor did not have an obvious difference between transgenic and WT plants (Fig. 4e).

### Subgroup analysis of the soluble sugar contents of plants under drought conditions

**Fig. 5:**
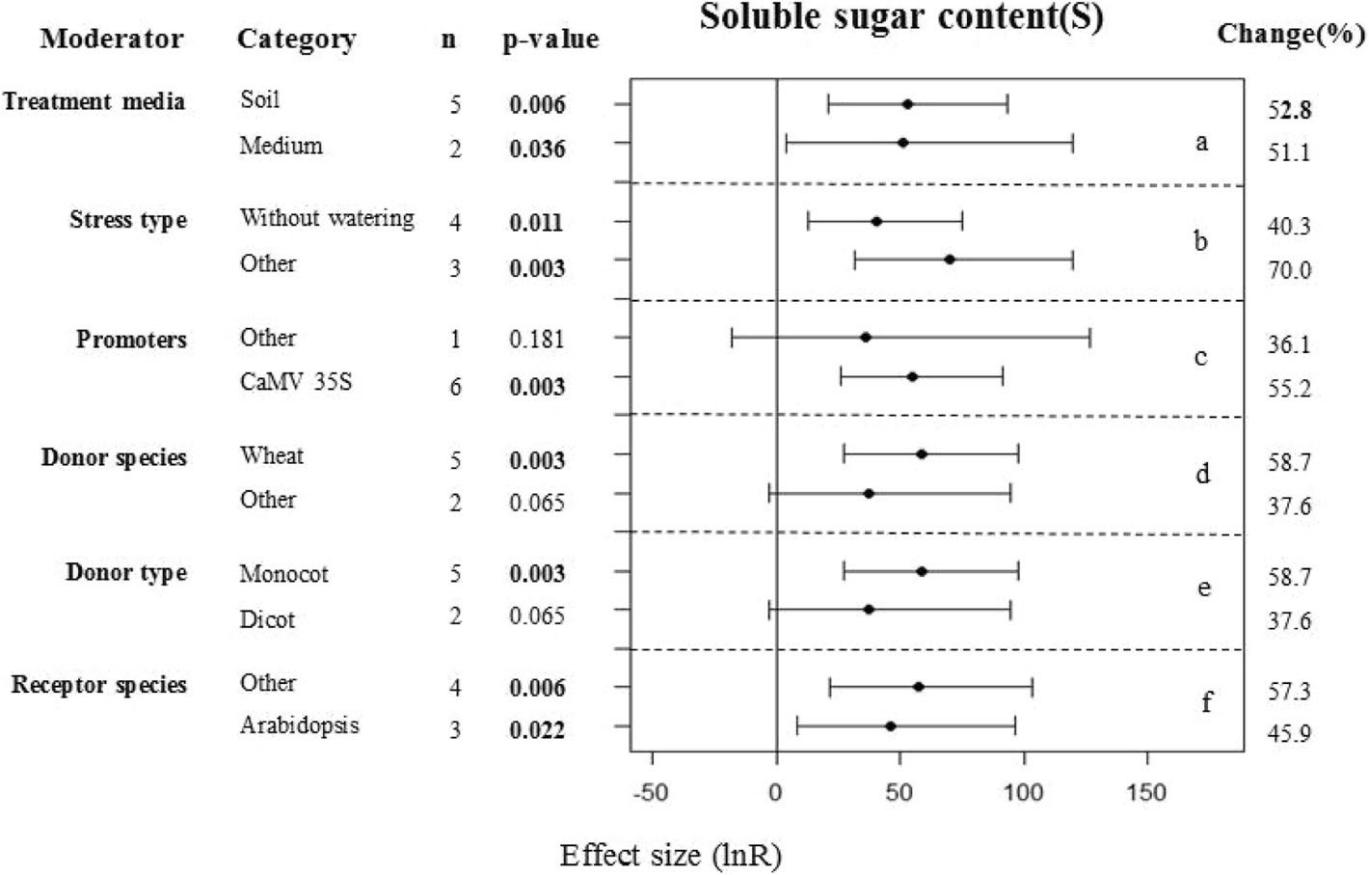
Subgroup analysis of the effects on soluble sugar content of plants under drought-stressed condition. There are six moderators on soluble sugar content in plants subject to drought (a-f) and category represents each moderator level

The soluble sugar contents of transgenic and WT plants under drought and control conditions were different (*p* = 0.09 and *p* = 0.199, respectively, Fig. 1). In contrast to the control conditions, use of different treatment media and recipient species with drought stress conditions increased the soluble sugar content in the transgenic plants over that of the WT plants (Figs. 5a, f and 8a, e). When promoters other than CaMV35S were used together with drought conditions, significant differences were found for the transgenic plants of the WT plants (Fig. 5c). Obviously differences were also detected when the gene donor species was a monocot (Fig. 5d, e). However, these differences were not significant under non-stressed conditions (Fig. 8b, c, d). Together, these results show that under drought conditions different types of stress can increase the soluble sugar contents in transgenic plants over those of WT plants (Fig. 5b).

### Subgroup analyses of the RWC and plant heights of plants under drought conditions

**Fig. 6:**
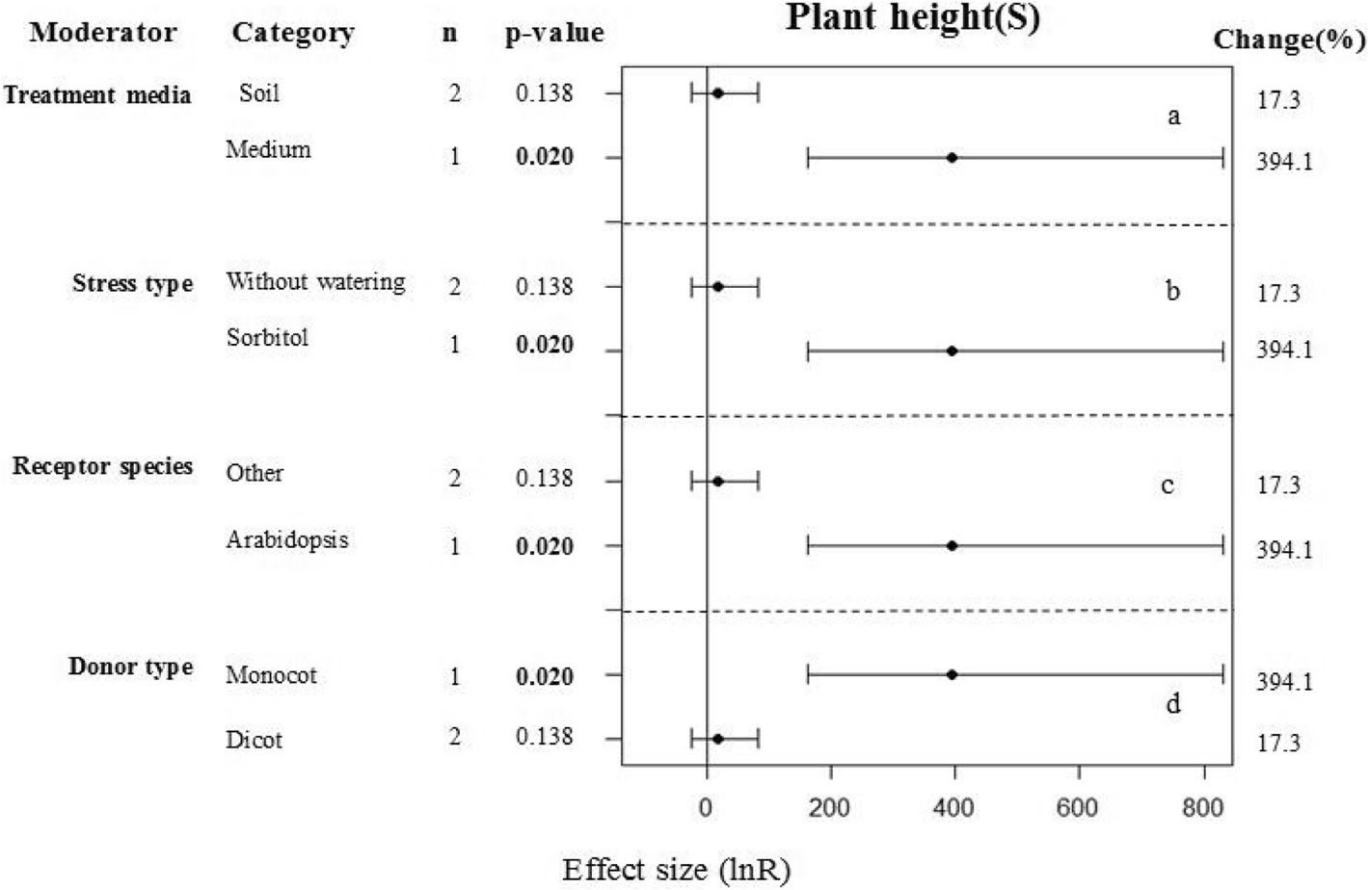
Subgroup analysis of the effects on plant height of plants under drought-stressed condition. There are for moderators on plant height in plants subject to drought (**a**-**d**) and category represents each moderator level

**Fig. 7:**
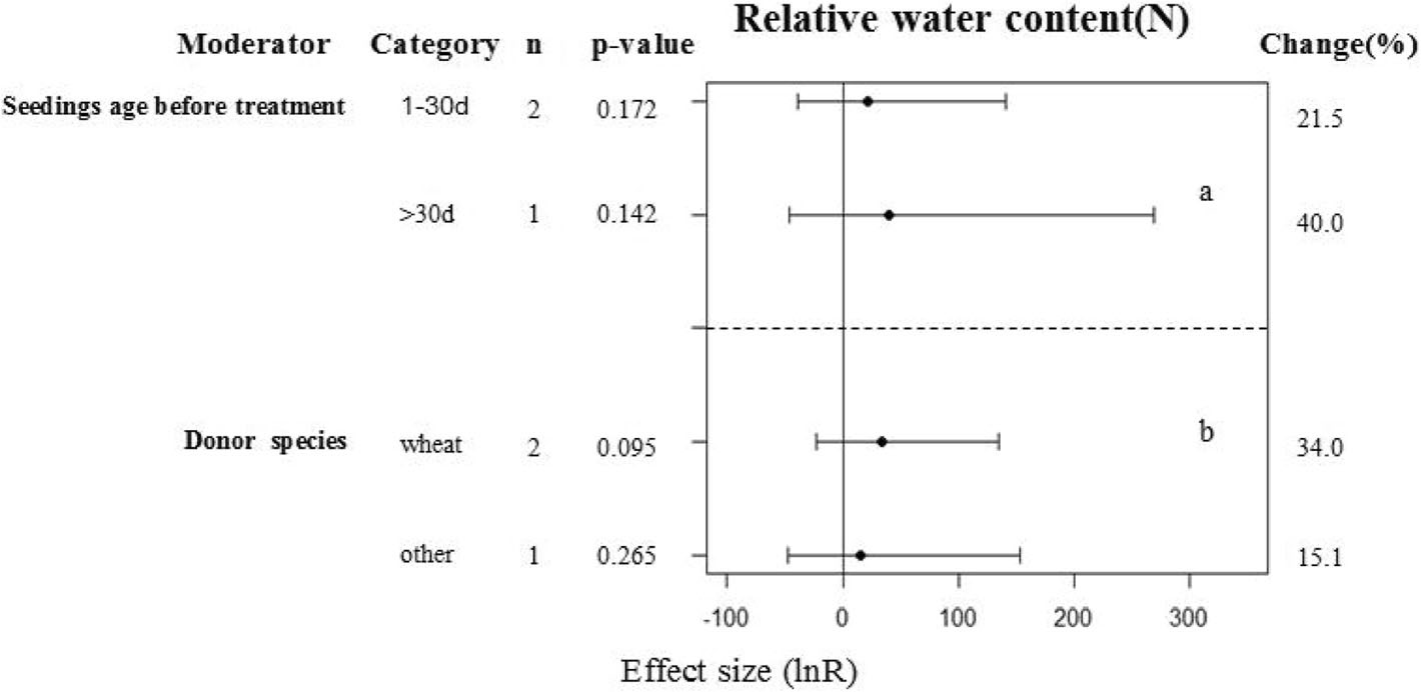
Subgroup analysis of the effects on RWC of plants under non-stressed condition. There are two moderators on relative water content in plants in control (**a**-**b**) and category represents each moderator level

The heights of the transgenic and WT plants under drought conditions was significantly different at a 90% confidence interval (*p* = 0.092, Fig. 1). The RWCs of the transgenic and WT plants under control conditions were significantly different at a 95% confidence interval (*p* = 0.009, Fig. 1). When the treatment media was solid medium, mannitol drought stress was applied, the recipient species was Arabidopsis, and the gene donor was a monocot species; the transgenic plants showed increased heights in comparison with the WT plants (Fig. 6a, b, c, d). Seeding age prior to treatment and the gene donor species used had no effect on the RWCs between the transgenic and WT plants under drought conditions (Fig. 7a, b).

### Heterogeneity

**Fig. 8:**
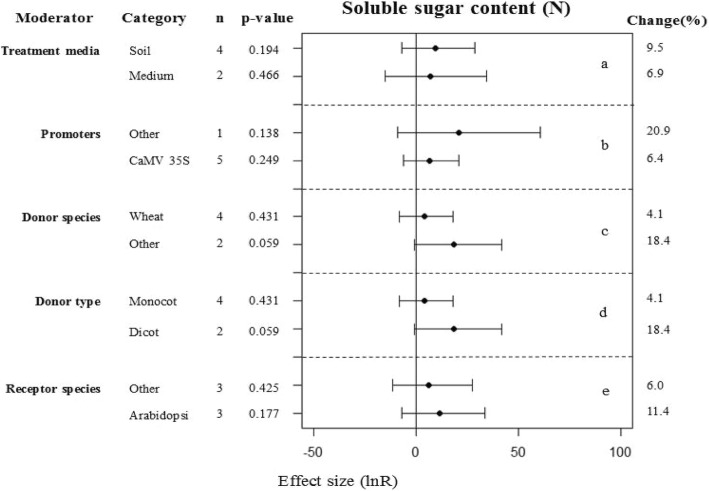
Subgroup analysis of the effects on soluble sugar content of plants under non-stressed condition. There are five moderators on soluble sugar content in plants in contrast (**a**-**e**) and category represents each moderator level

Tables 1 and 2 present the heterogeneity statistics of the summary effect sizes of the 16 parameters under different environmental conditions after data conversion (the log ratio of transgenic and WT plants). Additional file 2: Table S1 and Additional file 3: Table S2 (presented in supplementary materials) present the results before the data conversion (the difference between the transgenic and WT plants). The heterogeneity *p*-values were significant (*p* < 0.10) for 2 of the 16 summary effect sizes (Fig. 1), and these also had positive *I*^*2*^ values for the malondialdehyde content (*p* = 0.03, *I*^*2*^ = 49.8%) and the soluble sugar content (*p* = 0.00, *I*^*2*^ = 82.5%) for plants under non-stressed conditions (Table 1). Eleven of the summary effect sizes had *I*^*2*^ values of 0%, and three of the effect sizes had a small positive of *I*^*2*^-value but the *p*-value did not show significant heterogeneity (*p* > 0.10). When the true effect sizes differ between studies, the source of this real or true heterogeneity is often further investigated using moderator or subgroup analysis. We conducted a moderator analysis on the effect of the soluble sugar content which was detected as significantly different in Fig. 1a (*p* = 0.090), and the result of moderator analysis showed that transgenic and WT plants under normal conditions were significantly different at a near-to-90% confidence interval (Fig. 8). The heterogeneity of the *p*-values was significant (*p* < 0.10) and the summary effects also had positive *I*^*2*^ values for 3 of the 16 summary effect sizes presented in Fig. 1: root length (*p* = 0.00, *I*^*2*^ = 67.1%), malondialdehyde content (*p* = 0.02, *I*^*2*^ = 48.6%) and chlorophyll content (*p* = 0.00, *I*^*2*^ = 82.8%) of drought-stressed plants (Table 2). Twelve of the summary effect sizes had *I*^*2*^ values of 0%, and one effect size had a small positive *I*^*2*^ value but there was no significant heterogeneity (*p* > 0.10). We conducted a moderator analysis on the effects of root length that were detected as different in Fig. 1a, and found significant differences between the transgenic and WT plants under drought conditions at a near-to-90% confidence interval (Fig. 4). Before the data were converted, the heterogeneity *p*-values were significant (*p* < 0.10) for 2 and 13 of the summary effect sizes under normal and drought conditions, respectively. Due to the different measurement methods used across studies, the heterogeneity between studies was large. Use of the log ratio greatly reduced the heterogeneity of the study (Tables 1, 2); therefore, it was necessary to convert the data in our meta-analysis and the converted data were used in the integration analysis.

**Table 1:** Heterogeneity statistics for the 16 summary effect sizes under non-stressed condition after data conversed

Trait	Qt	P	*I*^*2*^ (%)
Survival rate	0.00	1.00	0.0
Stomatal aperture	0.01	1.00	0.0
Germination	0.03	0.98	0.0
Root length	7.12	0.93	0.0
Shoot fresh weight	0.11	1.00	0.0
Relative water content	0.01	1.00	0.0
Electrolyte leakage	7.37	0.29	18.6
Proline content	1.10	1.00	0.0
**Malondialdehyde content**	**21.91**	**0.03**	**49.8**
Chlorophyll content	13.31	0.15	32.4
**Soluble sugar content**	**28.51**	**0.00**	**82.5**
Plant height	0.00	0.95	0.0
H_2_O_2_ content	2.24	0.33	10.8
CAT activity	0.44	0.99	0.0
POD activity	0.06	1.00	0.0
SOD activity	0.02	1.00	0.0

*Qt* total heterogeneity, *P* probability that Qt was due entirely to sampling error and not to variation among true effects, *I*^*2*^ percentage of heterogeneity due to variation among true effects; Summary effect sizes showing significant heterogeneity among true effects (*p* ≤ 0.1) were shown in bold (Same for Table 2)

**Table 2:** Heterogeneity statistics for the 16 summary effect sizes under drought stressed condition after data conversed

Trait	Qt	P	*I*^*2*^ (%)
Survival rate	1.06	1.00	0.0
Stomatal aperture	0.05	1.00	0.0
Germination	0.07	1.00	0.0
**Root length**	**85.08**	**0.00**	**67.1**
Shoot fresh weight	6.78	0.66	0.0
Relative water content	0.21	0.99	0.0
Electrolyte leakage	10.15	0.52	0.0
Proline content	1.93	1.00	0.0
**Malondialdehyde content**	**29.18**	**0.02**	**48.6**
**Chlorophyll content**	**46.42**	**0.00**	**82.8**
Soluble sugar content	0.31	1.00	0.0
Plant height	2.75	0.25	27.4
H_2_O_2_ content	0.17	0.92	0.0
CAT activity	6.39	0.93	0.0
POD activity	1.99	1.00	0.0
SOD activity	.81	1.00	0.0

## Discussion

Tang et al. overexpressed *WRKY20* in alfalfa and found that the survival rate of transgenic plants was significantly higher (*p* < 0.01) than that of WT plants after 20 d of drought conditions followed by 7 d of re-watering [39]. Wang et al. [40] introduced *TaWRKY10* into tobacco and found that the survival rate of transgenic plants was much higher than WT plants after 3 weeks of drought treatment. Therefore, it appears that WRKY transcription factors play an important role in plants responses to drought stress. Our meta-analysis showed that with adequate water supply there was no difference in survival between transgenic and WT plants (*p* = 0.959). In contrast, under drought conditions, the survival rate of transgenic plants was higher than that of WT plants (*p* = 0.000). Most of the researchers in individual studies concluded that their transgenic plants had higher survival rates than the corresponding WT plants after rehydration. Our meta-analysis combined the results of many independent studies to give a confidence interval for this phenomenon. We found that most of the recipient species used to generate the transgenic plants included in our study were dicots (Fig. 3f). The reason for this is probably because model plants are usually dicots such as Arabidopsis or tobacco, and dicots have genomes that are relatively simple and have high transformation efficiencies. Therefore, these species are favored by the majority of researchers when exploring the gene functions. However, because of this favoritism, the results of our study have a risk of bias and care must be taken with extrapolating our results beyond these model plant species.

Plants accumulate large amounts of a variety of osmolytes in response to environmental stress [41]. These include soluble sugar, which is an important non-toxic osmoregulatory substance that is accumulated by plants under osmotic stress [42, 43]. The soluble sugar content of plants increases with an increase in water stress and this effect becomes more pronounced with increasing water stress. We found that increased soluble sugar content was the main effect associated with WRKY overexpression in transgenic plants and this was most pronounced under normal conditions (Fig. 2). The soluble sugar content, as an osmotic regulator, plays an important role in the resistance of plants to drought stress. Zhu et al. examined the effect of *VvWRKY30* overexpression on the accumulation of soluble sugars under drought stress and found that soluble sugar content increased in both transgenic and WT Arabidopsis plants, with the increase most pronounced in transgenic plants [44]. Ma et al. [45] overexpressed *TaWRKY146* in Arabidopsis. Although they found that there was no significant in the soluble sugar contents between transgenic and WT plants under normal growth conditions, they found significant differences in drought environment (*p* < 0.01). Other researchers have had similar results [39, 40, 46]. However, Niu et al. found that the soluble sugar contents were not significantly affected in transgenic lines in comparison with control Arabidopsis Col-0 plants exposed to sorbitol stress [47]. We speculate that these conflicting results may be primarily due to differences between the experimental treatments used (Fig. 5b). For example, we found that there was no significant difference in the soluble sugar accumulation between transgenic plants overexpressing WKRYs and WT plants under sorbitol stress (*p* = 0.066).

In general, drought stress inhibits root growth. This effect was can be measured in tolerant genotypes, although the effect is most prominent in sensitive genotypes, such as those of wheat [48]. Zhu et al. [44] overexpressed *VvWRKY30* in grape and found that after 7 d exposure to 300 mM mannitol the root growth of both the transgenic and WT plants were inhibited, although the effect was greatest in the WT plants. However, Arabidopsis plants overexpressing *VvWRKY30* that were grown on basal MS medium without any stress treatment had no significant differences in root growth from that of WT plants. Other researchers have drawn similar conclusions in other species, including cotton [49–51], wheat [40, 52], rice [53, 54] and soybean [39, 55]. These findings are also consistent with the results of our meta-analysis. We found that there were no differences in the root growth of transgenic and WT plants under control conditions (*p* = 0.5), but there were significant differences under drought conditions at a near-to-95% confidence interval (*p* = 0.181). These results suggest that WRKY transcription factors have a positive regulatory effect on the root lengths of transgenic plants and this is a relatively sensitive measurement parameter of plant responses to drought stress. However, not all researchers agree that WRKY transgenic plants have longer root lengths than WT plants under drought conditions [56]. Some consider that WRKY transcription factors have a negative effect on plant root lengths as part of their response to water stress (Fig. 4a). Through further analysis of these studies, we found that exposure to 1–5d of drought stress produced this negative effect. Therefore, we propose that the differential responses of WRKY transgenic plants to drought stress detected in different studies is related to the duration of drought stress. Further research is required to test this hypothesis.

When plants are subjected to drought stress they usually have reduced heights in comparison those grown under normal growth conditions. Tang et al. [39] and Niu et al. [47] introduced *WRKY20* into alfalfa and *TaWRKY2* + *TaWRKY19* into Arabidopsis, respectively. Both studies found that the heights of the transgenic plants were greater than those of WT plants under drought stress. However, Moon et al. [57] found no difference in plant height between transgenic and WT plants. This may be because the sample size in the latter study was smaller than those of the other two studies. From our meta-analysis, we concluded that that transgenic plants were much taller than WT plants under drought conditions at a 90% confidence interval (*p* = 0.092). However, when evaluating the drought tolerance of transgenic plants expressing WRKY genes, most researchers focus on morphological, physiological and biochemical indexes of plant growth at the seedling stage, and few consider traits such as plant height that are measured in older plants. Consequently, the number of studies that measured plant height that were included in our analysis was relatively small and the results were not significant. Further studies are required to obtain sufficient statistical power to analyze this parameter in the future.

Similar to plant height, the RWC parameter investigated in this study also has the problem that only a small number of studies were available. Drought stress retards plant growth through a cell physiology pathway. The plant RWC reflects the water retention capacity, and drought and dehydration stresses reduce the RWC. In contrast, plants with tolerance to these stresses may exhibit increased RWC [58]. Overexpression of *DnWRKY29* in tobacco and introduction of the maize *WRKY58* gene into rice [40, 46], significantly increased the RWCs of the transgenic plants under normal growth conditions. However, Sun et al. [59] found no differences in the RWCs of transgenic and WT plants under normal growth conditions. Therefore, even when investigating the same topic, different researchers may reach different or even opposite conclusions. These differences may be due to differences in the research methodologies and materials used. Our meta-analysis showed that there was a significantly difference in the RWC between transgenic and WT plants under control conditions at a 95% confidence interval (*p* = 0.009). However, it’s also due to the small number of studies included in our analysis, more studies are required to support this conclusion.

Besides the phenotypic indicators described above, there are other measured parameters that are sensitive to arid environments. These includes antioxidant defenses (SOD, POD and CAT activities and H_2_O_2_ content), cell membrane physiological indicators (stomatal aperture, electrolyte leakage and malondialdehyde content), compatible solute proline content, chlorophyll participation in photosynthesis (chlorophyll content) and morphological indices (germination, shoot fresh weight) [44, 56, 60]. However, none of these traits were found to be significantly different between transgenic and WT plants in our integration analysis and these parameters were not further investigated.

In our study, we found that the type of donor/recipient combination also played a vital role during transgenic plants adaption to drought stressed condition. A significantly greater positive moderator effect was observed when the gene donor and recipient were from the same genus than when the donor and recipient were from the different genus (Fig. 4c). Other researchers drew similar conclusions with us. Dong [38] carried out a meta-analysis of the effect for CBF/DREB TFs in plant adaption to drought stress, and they found that When the gene donors and recipients were composed of different genera, a greater increase in proline levels was observed than if the donor and recipient were from the same genus, no matter if the plants were subjected to non-stressed or stressed conditions. Ma [35] analysed the important role of cation/proton antiporter 1 genes on plants salt tolerance increasing by meta-analysis and found that when gene donors and recipients have been of different genera, the impact of transformation on root K^+^/Na^+^ ratio has been about twice as large as when donor and recipient were of the same genus, which can be beneficial to maintain root ionic balance under high-salt condition.

Many researchers [35, 36, 61] have carried out similar studies to ours that have focused on the differences between transgenic and WT plants under different environmental conditions. However, these studies have not investigated the differential performances of transgenic plants under different water conditions. To comprehensively evaluate the important role of WRKY transcription factors in plant resistance to drought stress, we not only compared the differences between transgenic and WT plants under drought and normal conditions, but we also examined the performance of transgenic plants grown in water-sufficient and water-deficient conditions. We found that9 of the 16 measured parameters had positive effects on plant growth under different water conditions, and 7 had negative effects. Eight of the 9 parameters with positive effects and 4 of the 7 parameters with negative effects were statistically significant (*p* < 0.05, Fig. 2). Our study shows that WRKY transcription factors play an essential role in plants responses to drought stress. Moreover, our findings will help future researchers to better understand the importance of the WRKY family genes in plants.

## Conclusions

As far as we all know, our study was the first one which examined WRKY transcription factors on plant adapting to drought stress by using meta-analysis. The main results of the present study showed that only one of these parameters was significantly different between transgenic and wild-type plants under drought and control conditions respectively, at a 95% confidence interval. However, due to the small sample size, more studies were needed here to further explore WRKY-overexpressed plants response to water stressed conditions. We also compared the performance of WRKY-overexpressed plans under drought and control environments. The meta-analysis revealed eleven of the parameters were obviously different in WRKY transgenic plants under drought and control conditions, suggesting that WRKY transcription factors play an important role in plant responses to drought stress. These findings also provide a theoretical basis for further study of the role of WRKY transcription factors in the regulation of plant responses to environmental stress.

## Methods

### Data collection

Endnote X7.1 was used to conduct a search using three electronic databases — PubMed, Web of Science and Springer Link — on 16th April 2018. The keywords (“WRKY transcription factors”) and (“drought” or “abiotic stress” or “water deficit”) and (“overexpression” or “over-express”) and (“crop” or “plant”) were used. After a quick appraisal of the title, abstract and keywords of the initial results, a total of 435 studies were selected that were then screened using the following criteria:

- publication written in English;
- publication between 2008 and 2018;
- includes at least some phenotypic evaluation parameters (such as plant height, germination, proline content, and malondialdehyde);
- the same research is not already published in a different database.
- it is not a review article or meeting report.

**Fig. 9:**
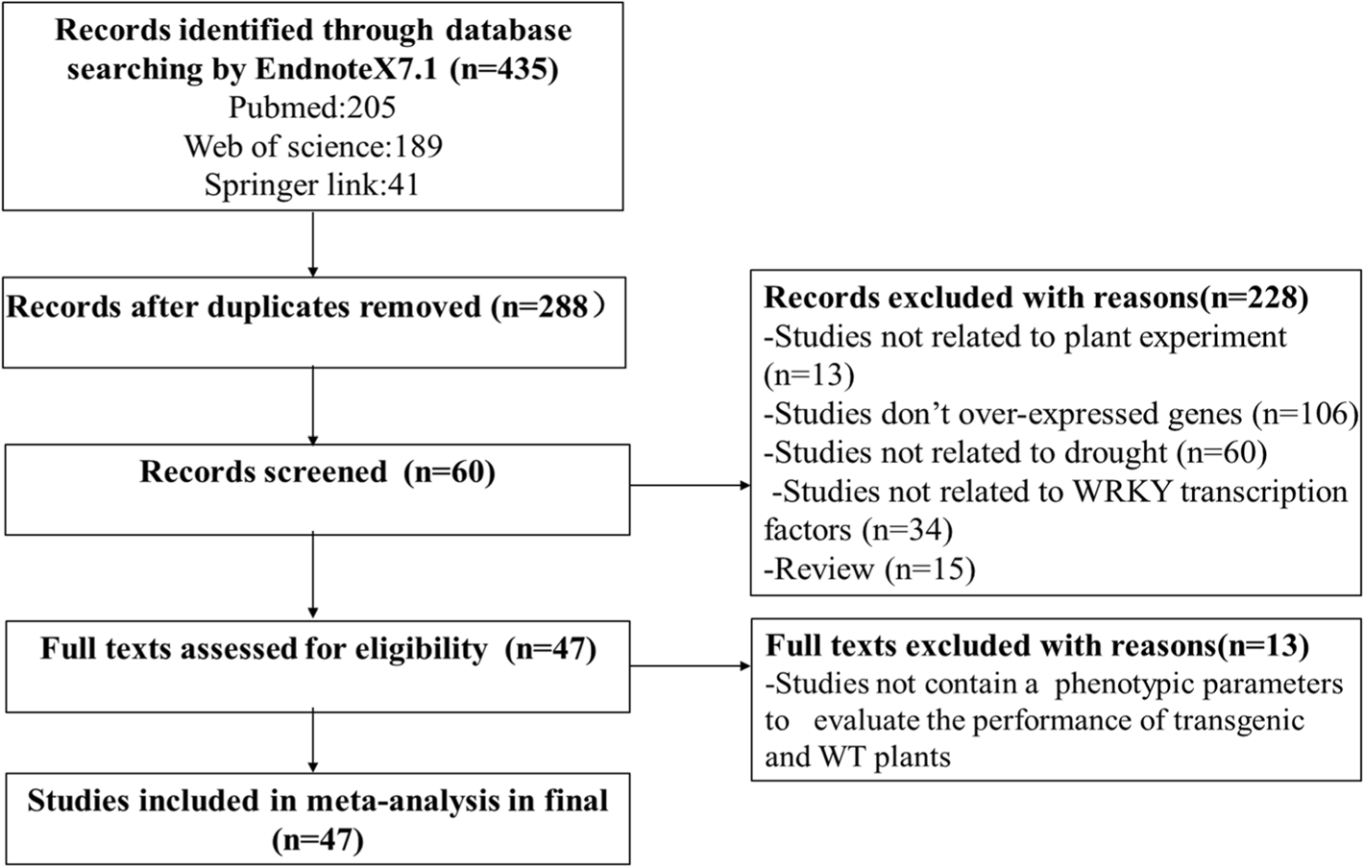
Study Selection Flow Chart

Of the 435 initial studies, 388 did not fully meet the above criteria: 13 studies were excluded because their data were unrelated to plants; 16 were review articles; 109 did not investigate overexpression of WRKY transcription factors; 38 were not related to WRKY transcription factors; 65 were unrelated to drought; and 147 were published in multiple databases. The remaining 47 studies that met the screening criteria were included in our meta-analysis (Fig. 9). Details of the primary studies are provided in Supplementary Materials (Additional file 1).

### Effect sizes and moderators

Several meta-analysis were conducted on parameters that were measured in published studies as adaptations to drought. Figure 1 lists a number of the response variables commonly measured in plants as adaptations to drought stress. To reduce variation in the meta-analysis, the natural logarithm (ln) of the response ratio (R), termed effect size, was calculated to measure the response of the plants to drought stress.

$$ \mathrm{Ln}\times \mathrm{R}=\left({\mathrm{X}}_{\mathrm{A}}/{\mathrm{X}}_{\mathrm{b}}\right) $$

Where X_A_ and X_B_ represents plants that were transformed with WRKY transcription factors and WT control plants, respectively. This formula describes the effect of overexpressing WRKY transcription factors or not under different environmental conditions. However, when the different effects of overexpressed transcription factors in plants under drought and control conditions were examined, X_A_ and X_B_ were used to represent transgenic plants subjected to drought and control conditions, respectively.

In addition to the effect size, information gathered from each study was recorded as a moderator that was used to evaluate for heterogeneity within the effect size. The moderators were derived from the following experimental variables: (1) experimental conditions (stress type, stress time, treatment medium); (2) experimental materials (the type and species of the WRKY gene donor and recipient, whether the gene donor and recipient were the same species and type, the seedling age before drought treatment, the type of promoter used); and (3) the number of WRKY genes being tested. Each moderator was set to at least two levels. If certain information was not presented in the original study, the moderator was set to null value and was not used in the classification.

### Meta-analysis

The nlum package of R Statistical software Version 3.1 (mixed-effects model) was used to calculate the grouped effect sizes [61]. If the 95% confidence interval of the forest plot overlaps with 0 (that is, the line of the single research level in the forest plot intersects with the vertical line), then the difference between the treatment group and the non-treatment group is considered as not significant. On the contrary, if there is no overlap, then there is 95% confidence that the difference between the two treatments is significant. The treatments are considered as significantly different from the control groups when *P* ≤ 0.05. The smaller the sample size of each trait, the larger the corresponding 95% confidence interval, and the longer the horizontal line shown in the forest plots. This reduces the reliability of the results. The closer the combined estimate of each trait is to the central axis (small dots on the horizontal line in the figure), the more likely it is that overexpression of the WRKY transcription factor does not affect that parameter. When the small dot is on the left side of the central axis this indicates that the WRKY transcription factor plays a negative regulatory role on the parameter and when it is to the right it represents a positive regulatory effect (these interpretations apply to Figs. 1, 2, 3, 4, 5, 6, 7, 8).

Heterogeneity was assessed using the Q statistic, a measure of weighted squared deviations. The Q statistic shows the presence of absence of heterogeneity, and is quantified using the descriptive index, *I*^*2*^, which estimates the ratio of true heterogeneity to total heterogeneity across the observed effect sizes [62, 63]. Total heterogeneity (Qt) is composed of the expected variation (the within-study heterogeneity, Qw, or sampling error) and the excess variation (the true heterogeneity in effect sizes between studies, Qm) [64]. Assumptions of homogeneity were considered valid when the *p*-values for the Q-test (chi-square test) for heterogeneity were more than 0.1, and were otherwise rejected. *I*^*2*^ is defined as (Qt − df)/Qt, where df represents the degrees of freedom in the expected variation and Qt − df, represents the true heterogeneity. Negative *I*^*2*^ values are set to zero so that *I*^*2*^ lies between 0 and 100%. A value of 0% indicates no true heterogeneity, while positive values indicate true heterogeneity in the data set. Larger *I*^*2*^ values reflect a larger proportion of the observed variation that is due to true heterogeneity between the studies [65].

If a single study contained multiple treatments, each treatment was treated as an independent study and so represented an individual unit in the meta-analysis [35, 65, 66]. This approach has been widely used in meta-analysis of plants [67–69]; in particular, for meta-analysis studies that examine the moderators in the original studies [70]. In total, our meta-analysis included a total of 158 independent studies derived from 47 published studies. Each of these are represented by the author + year of the published study and are suffixed with a lower-case letter for studies of plants subjected to drought stress, and with an upper-case letter for studies of plants subjected to normal conditions.

The means and sample sizes for the measured responses were gathered from each study for the treatment and control conditions. If there was no mention of the sample size, we defined it conservatively as *n* = 1. For studies that included multiple time points, the measurements taken at every time point were collected. GetData Graph Digitizer was used to determine the actual values when data were only presented in graph form (http://getdata-graph-digitizer.com) [38].

## Additional files

Additional file 1: The data of original studies on WRKY-overexpression. Details on the WRKY-overexpression studies used in the meta-analyses, including each of the moderators used for categorical analyses, the transgenic and WT means (X), sample sizes (N) and standard deviation (SD). (XLSX 106 kb)

Additional file 2: **Table S1.** Heterogeneity statistics for the 16 summary effect sizes under non-stressed condition before data conversed. Qt, total heterogeneity; P, probability that Qt was due entirely to sampling error and not to variation among true effects; *I*^*2*^, percentage of heterogeneity due to variation among true effects; Summary effect sizes showing significant heterogeneity among true effects (*p* ≤ 0.1) were shown in bold (Same for Table S2). (DOCX 17 kb)

Additional file 3: **Table S2.** Heterogeneity statistics for the 16 summary effect sizes under drought stressed condition before data conversed. (DOCX 17 kb)

## Data Availability

The datasets used for this study are available (included in Additional files).

## Abbreviations

CAT: Catalase
CHC: Chlorophyll content
EL: Electrolyte leakage
Ger: Germination
H_2_O_2_: Hydrogen peroxide
MDA: Malondialdehyde
PH: Plant height
POD: Peroxidase
Pro: Proline
RL: Root length
RWC: Relative water content
SA: Stomatal aperture
SFW: Shoot fresh weight
SOD: Superoxide dismutase
SSC: Soluble sugar content
SV: Survival rate
