# Supplementary material for: Meta-analysis of the effects of overexpression of WRKY transcription factors on plant responses to drought stress
Source: BMC Genet. 2019 Jul 26;20:63. doi: 10.1186/s12863-019-0766-4 (PMC6660937; doi:10.1186/s12863-019-0766-4)
Supplement: Supplementary file 2 — Table S1. Heterogeneity statistics for the 16 summary effect sizes under non-stressed condition before data conversed. Qt, total heterogeneity; P, probability that Qt was due entirely to sampling error and not to variation among true effects; I2, percentage of heterogeneity due to variation among true effects; Summary effect sizes showing significant heterogeneity among true effects (p ≤ 0.1) were shown in bold (Same for Table S2). (DOCX 17 kb) [file 12863_2019_766_MOESM2_ESM.docx]

| **Trait** | **Qt** | **P** | ***I^2^*(%)** |
| --- | --- | --- | --- |
| Survival rate | 0.00 | 0.99 | 0.0 |
| Stomatal aperture | 1.79 | 0.88 | 0.0 |
| Germination | 1.30 | 0.86 | 0.0 |
| Root length | 6.83 | 0.87 | 0.0 |
| Shoot fresh weight | 0.39 | 0.94 | 0.0 |
| Relative water content | 4.26 | 0.12 | 53.0 |
| Electrolyte leakage | 3.37 | 0.76 | 0.0 |
| **Proline content** | **25.49** | **0.00** | **65.0** |
| Malondialdehyde content | 5.17 | 0.74 | 0.0 |
| Chlorophyll content | 2.95 | 0.94 | 0.0 |
| **Soluble sugar content** | **12.02** | **0.02** | **67.0** |
| Plant height | 0.11 | 0.75 | 0.0 |
| H_2_O_2_ content | 2.81 | 0.25 | 29.0 |
| CAT activity | 3.73 | 0.59 | 0.0 |
| POD activity | 4.02 | 0.67 | 0.0 |
| SOD activity | 4.02 | 0.67 | 0.0 |
