# Supplementary material for: Meta-analysis of the effects of overexpression of WRKY transcription factors on plant responses to drought stress
Source: BMC Genet. 2019 Jul 26;20:63. doi: 10.1186/s12863-019-0766-4 (PMC6660937; doi:10.1186/s12863-019-0766-4)
Supplement: Supplementary file 3 — Table S2. Heterogeneity statistics for the 16 summary effect sizes under drought stressed condition before data conversed. (DOCX 17 kb) [file 12863_2019_766_MOESM3_ESM.docx]

| **Trait** | **Qt** | **P** | ***I^2^*(%)** |
| --- | --- | --- | --- |
| **Survival rate** | **711.1** | **0.00** | **98.0** |
| **Stomatal aperture** | **12.9** | **0.01** | **69.0** |
| Germination | 9.2 | 0.69 | 0.0 |
| **Root length** | **48.9** | **0.00** | **49.0** |
| Shoot fresh weight | 8.5 | 0.20 | 30.0 |
| **Relative water content** | **73.9** | **0.00** | **95.0** |
| **Electrolyte leakage** | **135.2** | **0.00** | **91.0** |
| **Proline content** | **176.9** | **0.00** | **65.0** |
| **Malondialdehyde content** | **115.0** | **0.00** | **90.0** |
| **Chlorophyll content** | **25.0** | **0.00** | **68.0** |
| **Soluble sugar content** | **35.8** | **0.00** | **92.0** |
| **Plant height** | **62.5** | **0.00** | **97.0** |
| H_2_O_2_ content | 4.0 | 0.13 | 50.0 |
| **CAT activity** | **31.8** | **0.00** | **59.0** |
| **POD activity** | **59.6** | **0.00** | **76.0** |
| **SOD activity** | **64.8** | **0.00** | **78.0** |
